# Mind Mapping Training’s Effects on Reading Ability: Detection Based on Eye Tracking Sensors

**DOI:** 10.3390/s20164422

**Published:** 2020-08-07

**Authors:** Ting Liu, Takaya Yuizono

**Affiliations:** 1 School of Knowledge Science, Japan Advanced Institute of Science and Technology, Nomi 923-1292, Japan; yuizono@jaist.ac.jp; 2 School of Foreign Languages, Dalian Polytechnic University, Dalian 116034, China

**Keywords:** eye tracking, eye movement indicators, mind mapping, reading ability

## Abstract

As a reading comprehension strategy, mind mapping has a positive influence on the development of students’ reading ability. However, how mind mapping affects reading ability has not been well documented. In this study, we used eye tracking sensors to explore mind mapping’s effects on reading ability. The participants were foreign language learning university students in Dalian city, China. One group received foreign language reading teaching integrated with mind mapping training (experimental group), and the other group received regular foreign language reading teaching (control group). We analyzed eye movement indicators, including fixation-related indicators (number of fixations, fixation frequency, and mean fixation duration), regression count, saccade amplitude, and pupil diameter. In addition, the analysis of heat maps and fixation trajectory maps, which are specific tool for visualization of eye movement data and intuitive analysis of reading process, were explained. The results show that the number of fixations, fixation frequency, mean fixation duration, and regression count in the experimental group were all lower than in the control group, and the pupil diameter was larger than in the control group. The heat map and fixation trajectory map show convergence, mostly focusing on the position of keywords and key sentences, with relatively large saccade amplitude and more information obtained by one gaze. Moreover, they had a higher skipping reading rate, which enhanced reading speed to obtain information accurately and quickly. These empirical results indicate that mind mapping training was an effective method for improving students’ reading ability.

## 1. Introduction

Reading ability is significant in foreign language learning [1]. Applying an appropriate reading comprehension strategy is crucial to the development of students’ reading ability [2]. Mind maps are made by beginning at the center and radiating out to successive levels of detail [3]. Mind mapping assists to fracture the text passage into sentences and words to make them easier to understand. Mind mapping is a manifest, hierarchical thinking process of knowledge visualization [3,4], which has a positive influence on deep learning, especially reading comprehension [5,6,7,8]. However, up to the present, the assessment method of mind mapping’s effectiveness on students’ reading ability has basically taken the form of traditional reading comprehension testing methods, which use the formats of “true or false, multiple choice and brief answers” to test article content comprehension. The reading ability is judged purely based on the scores obtained by students. This traditional way to test students’ reading abilities cannot visualize the cognitive processing of text understanding and the reading process directly [9]. To comprehensively test reading ability, it is necessary to analyze the cognitive process of reading effectively and study the real-time understanding process of discourse comprehension.

Linguistic psychology demonstrates that reading refers to the process of extracting information from the word system and then understanding the article through brain coding processing [10]. Nerves emanating from the cerebral cortex dominate eye movement, which is dominated by the brain and then reversed back to it. Eye movement is a biological signal, and its exploration may reveal lots of information which enables greater understanding of the biology and mechanisms of the brain [11]. Thus, eye movement can reflect our minds. Reading is a complex cognitive process; through eye movement analysis, the real-time language understanding cognitive processes are grasped accurately, and the reading process can be researched more in-depth [12,13]. Compared with the traditional comprehension testing methods, eye tracking technology provides more accurate and effective support. It is a research technology with high universality in the field of language understanding and is able to capture and analyze data of cognitive processing process in reading behavior and conduct comprehensive reading process analysis. It can not only provide real-time and visual measurement for language understanding but also continuously measure and record the entire process of reading comprehension.

The mind mapping training reading teaching strategy proposed in this study requires students to consciously find keywords and key sentences from the article and draft the structural framework of the whole article, which allows them to fully grasp the main content of the reading article and therefore realize the goal of quickly understanding the general idea of the article and therefore improve reading efficiency. Mind mapping is a cognitive process, thus it is of great practical significance to study how mind mapping training affects students’ reading process. In this study, the eye tracking research method was used to investigate eye movement characteristics of students in the process of reading; both students who accept mind mapping training as well as those who do not accept mind mapping training were included. The results show that there are differences in the eye movement indicators for the two groups. Participants of different reading levels have different cognitive processing levels during the reading processing, and the eye movement indicators can be used as a means of detection [14]. Data and insights culled from the findings were used to analyze the influence of mind mapping training on students’ cognitive processing, with the aim of providing objective data further supporting the hypothesis that mind mapping training can significantly improve reading ability.

## 2. Related Studies

Eye movement indicators can reflect cognitive process in the reading process, and this is an important research area of psycholinguistics and reading psychology [15]. Eye tracking sensors represent a human–computer interaction technique for recording online reading behavior, showing eye movement indicators during reading and providing sufficient data support for the reading comprehension process. It can be considered as a tool for biometric measures [16]. It was used to record eye movement indicators in real time, going on to map the eye movement indicators to the reading process that can effectively analyze the reading ability and provide a variable with psycho-ecological validity for reading research [17,18].

The reading process is the course of cognitive processing of visual information, and eye tracking is the most effective method of studying cognitive processing of visual information [19]. There is a definite corresponding relationship between reading process and eye tracking [20]; the eye tracking research method has an important role and position within the study of reading process. Reading ability has a significant influence on reading process; however, there is no fixed standard for the measurement or evaluation of reading ability. The study of the influence of reading ability on reading process can be attained by studying the differences of reading ability and cognitive processing. Reading ability is interrelated to the mode of eye movement, and through eye movement indicators the cognitive process of the differences in reading ability can be explained [13]. Through the study of eye tracking, we can understand the direction and times of eye movement during the process of reading, which can then provide a theoretical basis for the proposition of reasonable reading teaching methods to improve reading efficiency and learning effects [21,22,23]. Many previous studies have focused on researching eye indicators during the reading process, such as fixation-related indicators, saccade amplitude, regression, and pupil diameter [24,25]. Fixation-related indicators can best reflect the reading characteristics of participants, such as number of fixations, fixation frequency, and mean fixation duration. Saccade amplitude can accurately show the reading efficiency and cognitive processing ability of reading materials. Regression can effectively reflect the process of reprocessing the reading materials. Pupil diameter’s change is closely related to fixations, gaze, and other specific reading eye movements, which can analyze the psychological changes in people’s reading process [26,27,28]. Readers who have high speed in reading tend to produce fewer fixations when reading sentences and texts, along with a shorter fixation duration, a larger scanning range, and a smaller range of regression [29,30,31]. High level readers have shorter fixation duration and smaller regression count than ordinary readers [32,33]. Rayner et al. [14] found that, as the difficulty of the article increases, the cognitive processing becomes more complex and difficult. The saccade amplitude generally becomes shorter, while the regression count increases significantly, and the mean fixation duration becomes longer. Van Gerven et al. [34] stated that, the greater is the effort in cognitive processing, the greater is the change in scope of pupil diameter. Previous studies provide a basis for explaining the relationship between eye movement indicators and cognitive processing in reading. However, at present, in regards to research on the effect of mind mapping training in reading teaching methods, previous studies mostly use reading tests or questionnaires to evaluate reading ability or identify reading difficulties [5,6,7,8], while there are few studies on eye movement in the process of reading from the perspective of visual cognitive processing. As indicated above, these assessments only present the results of reading comprehension and cannot explain the real-time process of text comprehension, thus it is difficult to objectively measure and evaluate reading ability. As a step toward filling this gap, to make the research more scientific and the results more precise, this study adopted the method of using an eye tracking experiment to study the different eye movement characteristics between the experimental group and the control group of students in the reading process from the perspective of visual cognitive processing, which can then go towards explaining the effect of mind mapping training on reading ability.

## 3. Materials and Methods

### 3.1. Participants

The participants consisted of 40 university students from the school of Japanese studies at a university in Dalian city, China. All participants gave their informed consent for inclusion before they participated in the study. The experimental group (20 students, 13% male) and the control group (20 students, 14% male) had similar Japanese-language expertise skills. Both groups received a practical 8-week Japanese reading course. The experimental group’s reading teaching was integrated with mind mapping training. Students mainly learned to take a hierarchical or tree branch format to express the main idea of the article and keywords and key sentences of each paragraph. This reading comprehension strategy focuses on activating students’ divergent thinking to form an article structure frame, which clearly show the composition of the article and the specific details of each part. On the other hand, the control group was taught with a regular curriculum and the students learned in the traditional way. Both groups’ teaching was carried out by their current Japanese teachers with the same teaching materials.

To assess the reading ability improvement for the two groups, we first administered a reading comprehension test before and after the intervention. The reading comprehension test was created based on previous reading comprehension assessment instruments used in Japanese reading courses, which contained 46 test items with a full mark of 100. The difficulty levels of the pre-test and post-test were similar but not identical. The *t*-test results in Table 1 show that both groups received higher mean scores on the post-test, and their reading ability improved. However, the experimental group received higher mean scores than the control group did, and the differences between the pre-test and post-test was statistically significant (*t* = −2.12, *p* < 0.01). To the analyze the significant difference in reading ability improvement between the experimental group and the control group, an eye tracking experiment was conducted for comparing the eye movement characteristics of the two groups during their reading process.

### 3.2. Apparatus

**Figure 1:**
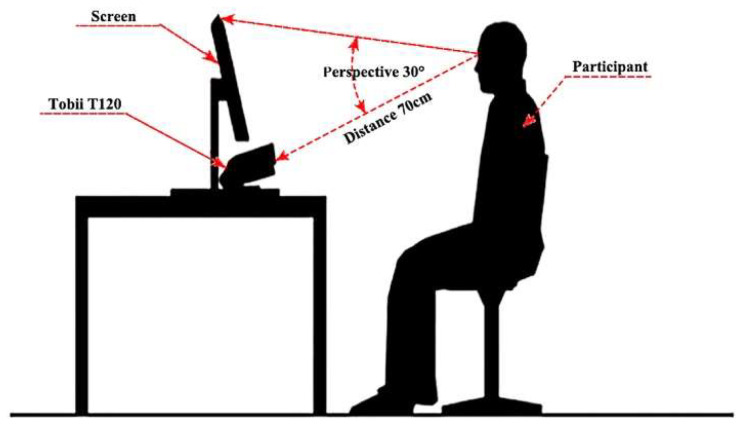
Schematic diagram.

**Figure 2:**
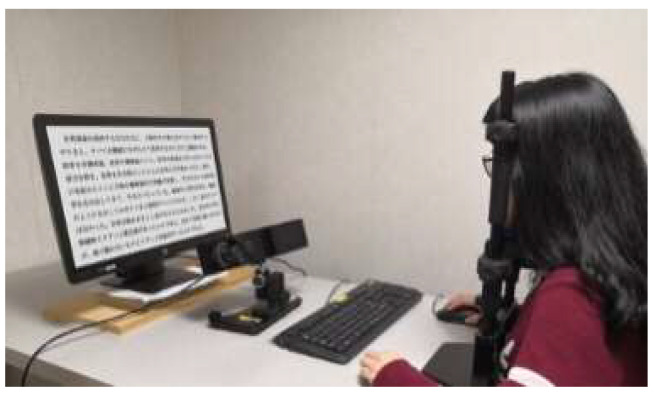
Experimental set-up.

The experiment used the Tobii T120 eye tracker, which is produced by Tobii in Stockholm, Sweden, with a sampling frequency of 1000 Hz and a resolution of the test machine display of 1024 × 768 pixels. The data of eye movement indicators were imported into Tobii Studio software. Figure 1 is a schematic diagram of the Tobii T120 eye tracker setup, and Figure 2 is the participant’s physical eye movement test. The participant’s eyes and the Tobii T120 eye tracker screen were always at a distance of 70 cm, which is approximately at a perspective of 30°.

### 3.3. Materials

The reading materials were the Japanese International Proficiency Test N2 exam reading comprehension simulation test materials. The number of words in each article is approximately 500, and the basic frame is 16 lines × 30 characters. The reading article contains Kanji, Hiragana, and Katakana, ensuring a comprehensive examination of Japanese reading ability.

### 3.4. Procedure

The procedures for obtaining eye movement indicators consist of the five steps as follows:

1. Give instructions to the participants about how to do the experiment.
2. Ask the participants to read four Japanese articles that are displayed randomly on a PC screen, one after another. The time given for reading is unlimited.
3. Record eye tracking data during reading.
4. Follow each article with two reading comprehension multiple-choice questions and a requirement to write down the keywords and key sentences to measure their reading comprehension rate and reading efficiency, which participants must do immediately after reading.
5. Analyze the obtained eye tracking data and other data (reading comprehension data).

### 3.5. Eye Movement Indicators

Among all kinds of eye movement indicators, fixation-related indicators, regression count, saccade amplitude, and pupil diameter are the most reported data in eye-tracking studies and recognized as primary indicators playing critical roles in identifying students’ reading abilities [24,25]. In addition, the heat map and fixation trajectory map can provide the visualization of eye movement indicators and intuitive analysis of reading process [35,36]. Therefore, in this study, these eye movement data were used for analysis.

#### 3.5.1. Fixation-Related Indicators

Fixation indicates the position where eye remains still for a certain period of time [37,38], which can reflect reader’s cognitive processing of reading materials [39]. Number of fixations, fixation frequency, and mean fixation duration are all general fixation-related indicators. Number of fixations is the number of gaze points while gazing at a certain area of interest (unit: number); fixation frequency is the number of gaze points per second (unit: times/s); and mean fixation duration refers to the average time that the eye remains still at each gaze point of a certain area of interest (unit: s). Compared to reading simple materials, reading difficult materials has significantly more fixations, and the fixation frequency and mean fixation duration would be higher [40].

#### 3.5.2. Regression Count

Regression refers to the reading process that move in the opposite direction to the reading materials [37,38]. The reason of regression eye movement may be that readers have difficulty in understanding the article [15]. Regression count is the number of regressions per second (unit: times/s).

#### 3.5.3. Saccade Amplitude

Saccade is the rapid movement between fixations [37,38]. Saccade amplitude refers to the distance at which the gaze point moves from one fixation to another (unit: °/s). The saccade amplitude can reflect the amount of information obtained in a single gaze, showing the reading efficiency and processing difficulty. The longer is the saccade amplitude, the more information would be obtained in a single gaze, making the reading speed faster [41].

#### 3.5.4. Pupil Diameter

Pupil diameter is an eye movement indicator used to infer the size of “cognitive processing” and “cognitive load”, which is often used in the field of education (unit: mm) [34]. The size of pupil diameter is closely related to the degree of psychological effort in information processing. When the psychological load is large, the range of pupil diameter will be larger [42,43]. In addition, it is also closely related to people’s emotions. For example, when people see things of interest, their pupil diameter will increase [42,43].

#### 3.5.5. Heat Map

**Figure 3:**
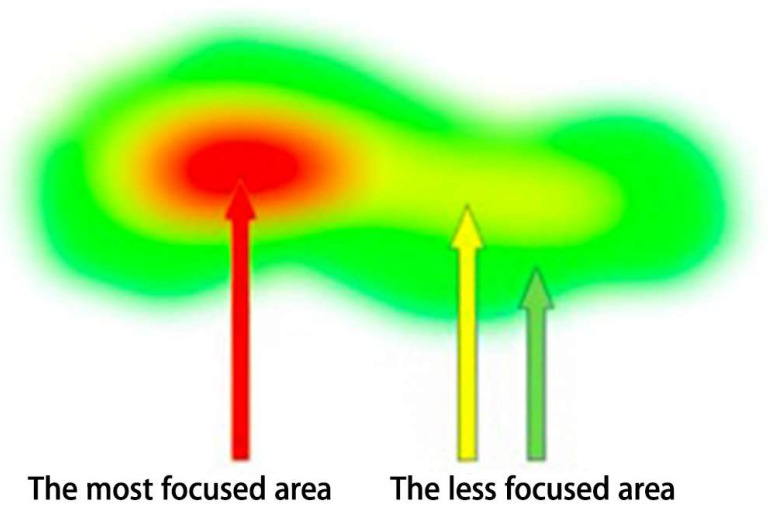
Schematic diagram of a heat map.

Heat map representing fixation duration and fixation location have been shown to be a better representation of visual processing [44]. It is a visual form that displays visual behavior features by superimposing eye movement data of multiple participants, thus reflecting the distribution of the visual trajectory and visually displaying key areas of visual attention on the interface. In addition to representing the concentration trend and dwell time of watching on the same page, it can also express eye movement patterns as they are superimposed on the same interface [45,46]. Figure 3 is a schematic diagram of a heat map. The focused area is represented by a spectrum of red–yellow–green, with red showing the area of most focus and green showing the area of least focus.

#### 3.5.6. Fixation Trajectory Map

**Figure 4:**
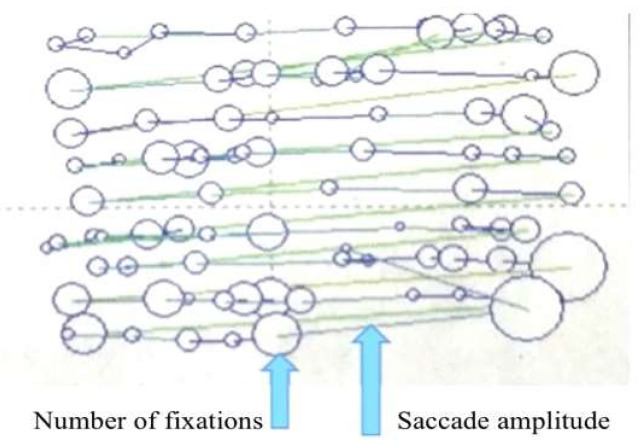
Schematic diagram of a fixation trajectory map.

Figure 4 is a schematic diagram of a fixation trajectory map. Circles in the fixation trajectory map indicate the number of fixations; the size of the circle indicates the fixation duration; and the lines between the circles indicate the saccade amplitude. By examining the fixation trajectory map, the complete reading process can be observed.

### 3.6. Reading Comprehension Indicators

To measure reading comprehension rate and reading efficiency, each article was followed by questions that participants needed to answer after eye tracking. Reading comprehension indicators were used for analysis, which are divided into four aspects: reading time, reading speed, reading comprehension rate, and reading efficiency [47,48]. Reading time refers to the time (in min) spent reading the entire article; reading speed refers to the number of words (words/min) read per minute; reading comprehension rate refers to the number of questions answered correctly divided by the total number of questions; and reading efficiency refers to reading speed times the reading comprehension rate.

## 4. Findings and Discussions

### 4.1. Eye Movement Indicators Analysis

**Figure 5:**
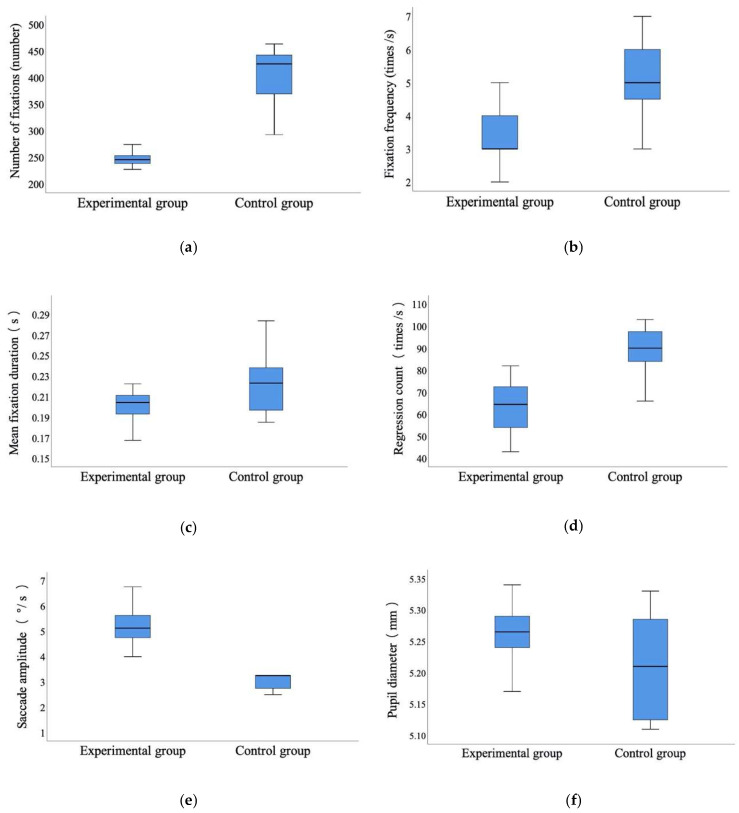
Statistical comparison of number of fixations, fixation frequency, mean fixation duration, regression count, and saccade amplitude for the two groups. (**a**) number of fixations; (**b**) fixation frequency; (**c**) mean fixation duration; (**d**) regression count; (**e**) saccade amplitude; and (**f**) pupil diameter.

Based on the statistical comparison in Figure 5, *t*-test was conducted (Table 2). The results show that the number of fixations, fixation frequency, mean fixation duration, and regression count were all significantly lower for the experimental group compared to the control group (number of fixations, *t* = 13.375, *p* < 0.001; fixation frequency, *t* = −4.094, *p* < 0.001; mean fixation duration, *t* = 2.663, *p* < 0.05; regression count, *t* = 8.062, *p* < 0.001). In terms of the saccade amplitude, the experimental group was significantly higher than the control group (*t* = −6.473, *p* < 0.001). The pupil diameter in the experimental group was larger than that in the control group, although there were no significant differences (*t* = 1.961, *p* > 0.05).

These eye movement indicators can reflect the understanding and information processing of the article when reading. From the perspective of fixation, a high number of fixations and fixation frequency indicate the difficulty in interpreting the fixated information [49], and shorter mean fixation duration indicates a higher work efficiency [50]. As skills improve, more information can be extracted around the point of fixation, making eye movements more effective overall [39]. Readers who have difficulty in understanding tend to re-read and have more regressions [51,52]. Saccade amplitude can reflect task difficulty; longer saccade amplitudes are more exploratory [53]. Pupil diameter provides a quantitative index for measuring the psychological load of information processing and can be used as an indicator of cognitive processing intensity. In general, the greater the effort of cognitive processing is, the greater the memory load is, and thus the greater the pupil diameter will be [34].

The number of fixations and fixation frequency were lower in the experimental group than in the control group. The number of fixations and fixation frequency can reflect the comprehension and information processing of the subjects, which indicates that the experimental group can understand the reading materials easily.

The mean fixed duration in the experimental group was shorter than the control group. The length of the mean fixed duration is related to the semantic extraction of the observed words. The mean fixed duration of the experimental group was 0.20 s, which can show that the main content of the reading materials can be extracted in a short time and the reading speed can be improved.

The regression count of the experimental group was significantly less than of the control group. Generally, regression is produced when there are difficulties and errors in understanding the reading content. The regression count of the control group was 90 times/s, which could infer that there were some difficulties in extracting and processing the information of reading materials, which requires repeated processing to achieve the goal of understanding the content of reading materials.

Saccade amplitude can reflect reading efficiency and processing difficulty. The saccade amplitude of the experimental group was significantly larger than that of the control group, which indicates that the experimental group could obtain a larger amount of information from the reading material and had a higher level of reading efficiency, while the saccade amplitude of the control group was small, indicating difficulty in processing reading materials.

The pupil diameter of the experimental group was larger than that of the control group. This indicates that the experimental group paid more attention to the information processing when reading materials, and their effort of cognitive processing was higher than that of the control group. In addition, it is possible that, after mind mapping training, the experimental group was more confident in their reading comprehension and had positive emotions regarding the completion of the reading task. The difference between the two groups did not reach a significant level, which may be due to the fact that the reading materials were not difficult and did not cause excessive psychological load on either group.

The reason the two groups have the above differences in these eye movement indicators is that the cognitive processing level of students with different reading ability will be different during the reading process. Mind mapping training can effectively improve the reading comprehension rate, while reducing the total reading time, the number of fixations, fixation frequency, mean fixation duration, and regression count and increase the saccade amplitude and pupil diameter, which can help the students to complete reading comprehension more quickly and accurately.

### 4.2. Heat Map Analysis

**Figure 6:**
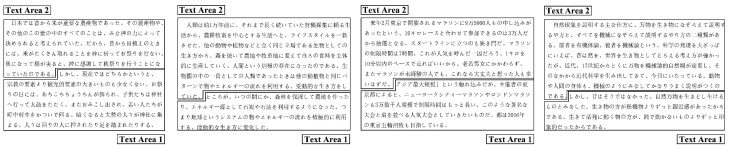
Text Areas 1 and 2 in four Japanese reading articles.

The visual focused areas were compared by detecting the mean fixation duration and fixation location in heat maps. As presented in Figure 6, in this experiment, four Japanese reading articles were delineated into two specific visual areas: Text Area 1 (first specific visual area)—the area of the “keywords and key sentences” section—and Text Area 2 (second specific visual area)—the area of other parts.

**Figure 7:**
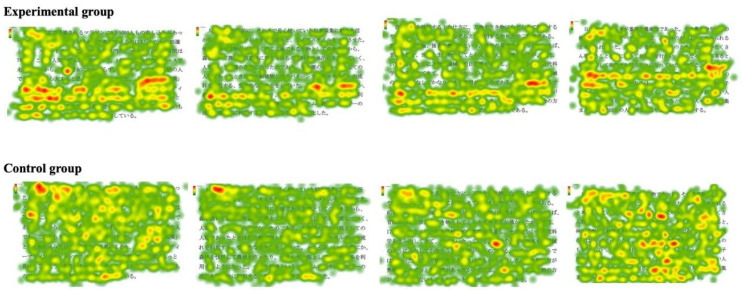
Heat map of the two groups in four Japanese reading articles.

Figure 7 depicts heat maps of all participants for the four Japanese reading articles. For the heat maps of the experimental group, the red hotspot particles were large, and the hotspots were convergent, mainly concentrated in Text Area 1, the keywords and key sentences, and appeared rarely in other parts of the material. In terms of the heat maps of the control group, the red hotspot particles were discrete, and the hotspot areas were less concentrated.

**Figure 8:**
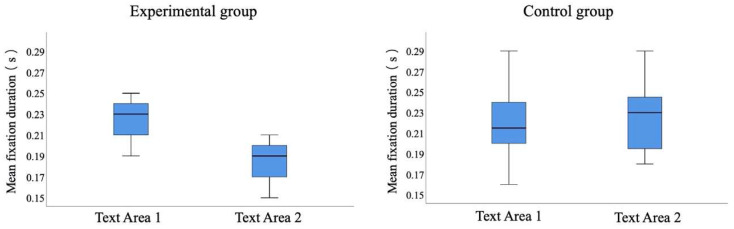
Statistical comparison of mean fixation duration on Text Areas 1 and 2 for the two groups.

The results are shown in Figure 8 and Table 3. The experimental group’s mean fixation duration on Text Area 1 of four Japanese reading articles were significantly higher than that on Text Area 2 (*t* = 6.543, *p* < 0.001). On the other hand, there were no significant differences in the control group’s mean fixation duration on Text Areas 1 and 2 of four Japanese reading articles (*t* = −0.526, *p* > 0.05).

The experimental group’s most focused areas were the keywords and the key sentences, and the control group’s reading process was fragmented. The results show that different reading teaching methods had different reading effects, and they affected the students’ most focused areas on the reading materials. Through mind mapping, the students could create associations on keywords and key sentences, establish memory links, and use relevant hierarchical diagrams to integrate the language knowledge in the reading materials [6,7]. The students in the experimental group were able to grasp the keywords and key sentences for reading, which promoted the grasp of the structure and the understanding of the main idea of the article. In the case of the control group, the students were taught in the traditional reading teaching classroom, and teacher-centered teaching was conducted, which did not require students to do the practices to find key words and key sentences in the reading materials. Thus, the eye movement characteristics on the heatmaps for the two groups were also different.

### 4.3. Fixation Trajectory Map Analysis

**Figure 9:**
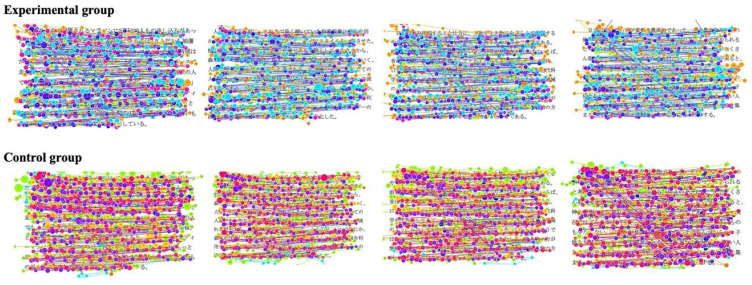
Fixation trajectory map of the two groups in four Japanese reading articles.

Figure 9 is the fixation trajectory map for all participants in four Japanese reading articles.

**Figure 10:**
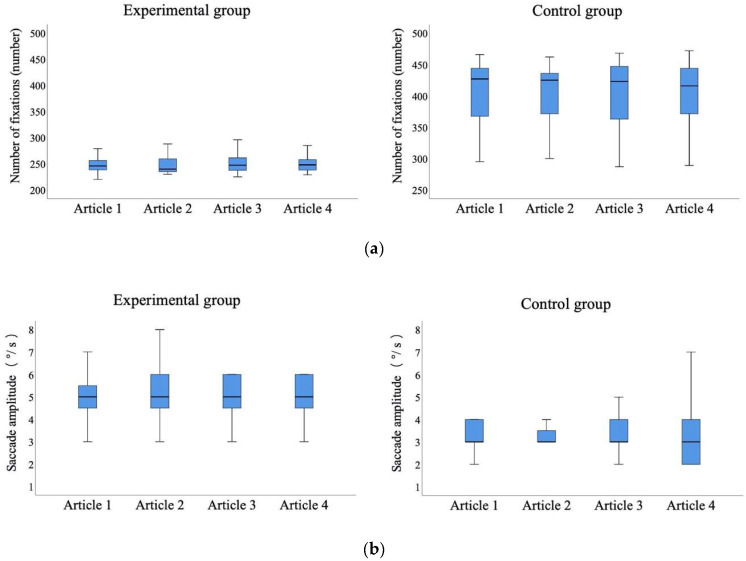
Statistical comparison of the number of fixations and saccade amplitude on four Japanese reading articles for the two groups: (**a**) number of fixations; and (**b**) saccade amplitude.

Number of fixations and saccade amplitude results for the two groups are shown in Figure 10 and Table 4. It indicated that the experimental group’s gaze moved more quickly and the number of fixations was lower: the average number of fixations of the four Japanese reading articles was 250.91. The saccade amplitude was larger: the average saccade amplitude of four Japanese reading articles was 5.06 °/s. In contrast, the control group read each sentence (from first to last) carefully. The speed of eye movement between words and sentences was slow and the number of fixations was higher: the average number of fixations of the four Japanese reading articles was 404.70. The saccade amplitude was smaller: the average saccade amplitude for the four Japanese reading articles was 3.34 °/s.

As mentioned in the *t*-test of Table 2, the number of fixations was significantly lower for the experimental group as compared to control group (*t* = 13.375, *p* < 0.001). In terms of the saccade amplitude, the experimental group was significantly higher than the control group (*t* = −6.473, *p* < 0.001). The results show that the experimental group’s reading process was relatively smooth, and the overall grasp of the article was good, which could save reading time and improve reading speed. On the other hand, the control group had some difficulty in reading the article, which would lead to increasing reading time and decreasing reading speed.

**Figure 11:**
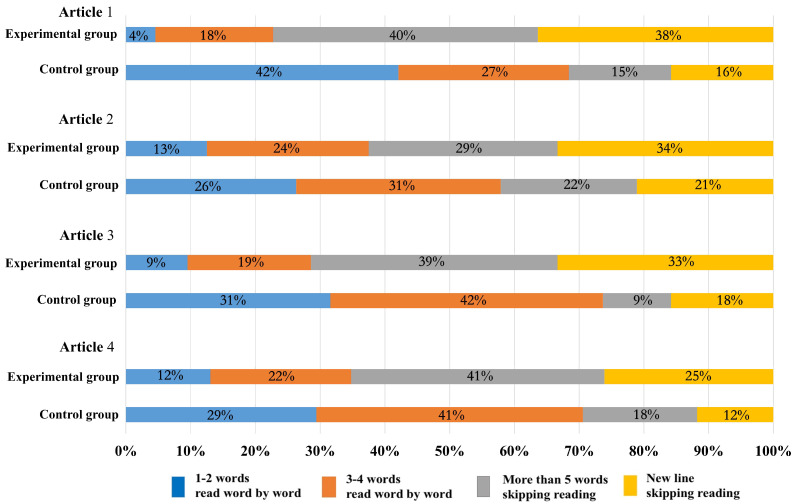
Proportion of skipping reading in the two groups.

During the reading process, people obtain information through fixations and transferred the fixations through saccades. The saccade amplitude changed due to the difficulty of reading materials. Saccade amplitude in fixation trajectory map indicates the skipping phenomenon. The greater is the saccade amplitude, the more words are skipped [54]. During the reading of Japanese articles, when gazing, readers usually see between two and five (mostly three and four) characters [55]. In this experiment, skipping was defined as five words or more sight moving, or new line sight moving. Figure 11 shows the proportion of the skipping phenomenon in the entire line of sight movement data during the reading process of four Japanese articles in both groups. The results show that the skipping rate in the experimental group was high, at almost 70%, while, in the control group, it was low, at almost 30%. Skipping depends on the difficulty level of the entire article; the lower is the difficulty level of the reading material, the higher is the skipping rate [54]. It indicated that the difficulty level of the reading materials for the two groups was different, and the two groups showed different skipping rate. Reading materials were relatively less difficult for the experimental group, which may be related to the mind mapping training effects. Through mind mapping training, the attention and time used on the reading unit can be reasonably allocated, and key points can be selected for reading to improve students’ reading efficiency [5], which will reduce the reading processing difficulty and make reading process more smooth to a certain extent. Therefore, the saccade amplitude of the experimental group was larger, and the skipping rate was higher than that of the control group.

### 4.4. Reading Comprehension Indicators Analysis

To further investigate the reading ability of the two groups, reading comprehension indicators analysis was conducted. As presented in Table 5, the *t*-test results show that there were significant differences in the reading comprehension indicators between the two groups. By comparing with the control group, in the experimental group, the reading time was significantly shorter (*t* = 6.075, *p* < 0.001); the reading speed was significantly faster (*t* = −5.674, *p* < 0.001); and the reading comprehension rate and reading efficiency were significantly higher (*t* = −3.035, *p* < 0.01; *t* = −5.822, *p* < 0.001).

The results further confirm the considerable reading effect of mind map training, which can save reading time, improve reading speed, enhance reading comprehension rate and reading efficiency, and thus promote students’ reading ability to a certain extent. Mind mapping training can activate students’ reading skills to form a clear article structure frame in head. It can clearly show the structure of articles, so that students can more clearly understand and grasp the specific details of each paragraph and analyze the structure and content of the article in detail. Through mind mapping, students will have a clear grasp of the overall structural context and layout of the article, as well as deepen their understanding of the content of the article and obtain detailed information efficiently. It can improve their ability to analyze micro-details of the article.

## 5. Conclusions

By eye tracking analysis, the reading process can be inspected in-depth. In this study, we used eye tracking sensors to record eye movement indicators in real time, which provides a quantitative assessment and data evidence of mind mapping’s effectiveness on reading ability. The results suggest that there are significant differences between the experimental group and control group’s eye movement indicators during the reading processes. Their different eye movement indicators reflect their different states as parts of the process of information input and information extraction. Students in the experimental group were found to understand the reading materials more promptly, showing fewer fixations, fewer regressions, longer saccades, and larger pupil diameter. Previous studies on mind mapping training effectiveness mainly focused on the traditional assessment of reading comprehension testing methods. This study applied eye movement indicators to investigate cognitive comprehension process, so as to measure and evaluate mind mapping training effectiveness on reading ability more comprehensively. However, certain limitations should be noted. First, although the sample size was sufficient to clarify the effectiveness of mind mapping training to improve students’ reading ability, it is still a small number and low gender balance for the generalization of eye tracking results. In the future, it is necessary to conduct an experiment with larger number and higher gender balance of participants and to analyze the results further. Second, this study only applied six eye movement indicators to describe and explain the reading process. It may give limited implication to the correlation between the improvement of reading ability and the eye movement indicators. More eye movement indicators could be considered for further investigation and verification, such as refixation rate, landing position, and first fixation duration. Third, in future work, we look forward to recognizing students’ cognitive and emotional status according to their eye movement indicators in order to have a more comprehensive and in-depth understanding of the cognitive process of reading.

## Figures and Tables

**Table 1:** The comparison of reading ability improvement for the participants.

Item	Group	Pre-Test		Post-Test		*t*	*p*
		M	SD	M	SD		
Reading comprehension test	Experimental group	78.31	2.35	84.65	2.52	−2.12 **	0.006
	Control group	78.22	2.76	80.20	2.68	−1.85	0.064

M–Mean; SD–Standard Deviation; * *p* < 0.05; ** *p* < 0.01; *** *p* < 0.001.

**Table 2:** The *t*-test results of eye movement indicators for the two groups.

Eye Movement Indicators	Experimental Group		Control Group		*t*	*p*
	M	SD	M	SD		
Number of fixations	250.91	19.16	404.70	47.78	13.375 ***	0.000
Fixation frequency	3.60	1.19	5.10	1.12	−4.094 **	0.001
Mean fixation duration	0.20	0.01	0.22	0.03	2.663 *	0.015
Regression count	63.15	11.80	90.00	8.94	8.062 ***	0.000
Saccade amplitude	5.06	0.93	3.34	1.01	−6.473 ***	0.000
Pupil diameter	5.25	0.06	5.21	0.08	1.961	0.065

M–Mean; SD–Standard Deviation; * *p* < 0.05; ** *p* < 0.01; *** *p* < 0.001.

**Table 3:** The *t*-test results of mean fixation duration for the two groups.

Group	Text Area 1		Text Area 2		*t*	*p*
	M	SD	M	SD		
Experimental group	0.223	0.023	0.183	0.017	6.543 ***	0.000
Control group	0.222	0.032	0.224	0.034	−0.526	0.605

M–Mean; SD–Standard Deviation; * *p* < 0.05; ** *p* < 0.01; *** *p* < 0.001.

**Table 4:** Number of fixations and saccade amplitude for the two groups.

**Number of fixations**	**Group**	**Article 1**	**Article 2**	**Article 3**	**Article 4**	**Average**
	Experimental group	250.50	248.70	252.85	251.60	250.91
	Control group	405.60	404.95	402.55	405.70	404.70
**Saccade amplitude**	Experimental group	4.95	5.15	5.15	5.00	5.06
	Control group	3.40	3.25	3.45	3.25	3.34

**Table 5:** The *t*-test results of reading comprehension indicators for the two groups.

Reading Comprehension Indicators	Experimental Group		Control Group		*t*	*p*
	M	SD	M	SD		
Reading time	1.25	0.25	1.90	0.34	6.075 ***	0.000
Reading speed	250.90	52.87	163.52	30.89	−5.674 ***	0.000
Reading comprehension rate	0.81	0.14	0.66	0.16	−3.035 **	0.007
Reading efficiency	200.34	49.03	109.26	38.67	−5.822 ***	0.000

M–Mean; SD–Standard Deviation; * *p* < 0.05; ** *p* < 0.01; *** *p* < 0.001.
